# Efficient Photolysis of Multidrug‐Resistant Polymicrobial Biofilms

**DOI:** 10.1002/advs.202407898

**Published:** 2024-12-21

**Authors:** Yongli Li, Yan Dong, ZhengKun Zhang, Zuan‐tao Lin, Chen Liang, Mei X. Wu

**Affiliations:** ^1^ Wellman Center for Photomedicine Massachusetts General Hospital Department of Dermatology Harvard Medical School 50 Blossom Street Boston MA 02114 USA; ^2^ Institute of Precision Medicine the First Affiliated Hospital Sun Yat‐sen University Guangzhou 510080 P. R. China; ^3^ Department of Stomatology Xuanwu Hospital Capital Medical University No. 45 Changchun Street, Xicheng District Beijing 100053 P. R. China

**Keywords:** antimicrobial, biofilm matrix, photolysis, polymicrobial biofilms, wound infection and cleansing

## Abstract

Chronic wounds are prone to infections with multidrug‐resistant bacteria, forming polymicrobial biofilms that limit treatment options and increase the risk of severe complications. Current cleansing options are insufficient to disrupt and remove tenacious biofilms; antibiotic treatments, on the other hand, often fall short against these biofilm‐embedded bacteria. This study explores an non‐antibiotic approach that extends beyond conventional porphyrin‐based phototherapy by using blue light (BL) in conjunction with ferric ions (Fe(III)) to disrupt and eradicate biofilms. The dual not only degraded biofilm extracellular polymeric substances (EPS) in mono‐species and polymicrobial biofilms by specifically targeting carboxyl‐containing polysaccharides within the matrix but also exhibited broad‐spectrum antimicrobial activity by affecting key components of the outer membrane and cell wall. Bacteria, such as *K. pneumoniae*, with compromised EPS after photolysis, demonstrated increased susceptibility to macrophage phagocytosis. Disruption of the polymicrobial biofilm structure also enhanced the bacterial susceptibility to bactericidal drugs. Treating wounds infected by mixed‐species biofilm in diabetic mice demonstrated a substantial reduction in bacterial colonization and improved tissue repair. The BL‐Fe(III) modality offers a safe, efficient alternative for managing chronic wound infections, making it ideal for repeated, non‐invasive use at home, especially in resource‐limited areas.

## Graphical abstract

Blue light (BL) combined with Fe(III) offers a novel anti‐biofilm mechanism that outperforms canonical porphyrin‐based phototherapy. The BL‐Fe(III) approach simultaneously disassembles biofilms and inactivates bacteria regardless of BL susceptibility (i). Bacterial loads at the wound will be reduced by follow‐up wound cleansing (ii), antibiotic killing (iii), and host clearance (iv).

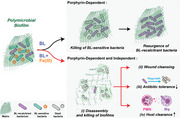

## 1 Introduction

Chronic wounds, particularly diabetic wounds (DW), are highly susceptible to bacterial infections due to the chronic breach of skin and impaired host immunity, leading to cellulitis and lethal sepsis if left unchecked.^[^ ^1^ ^]^ Multidrug‐resistant microbes and polymicrobial biofilms are commonly found in DW infections, which greatly limit treatment options and severely delay wound healing. These biofilms protect the embedded microbes, enhancing their survival and facilitating gene exchanges. They also protect the bacteria from immune clearance, making the biofilms extremely challenging to control. Currently, the standard treatment requires constant debridement of the wounds and physical removal of the infected tissues in a hospital setting by skilled professionals. Despite such efforts, wound infections can still rapidly relapse and deteriorate between hospital visits. Consequently, an alarming fifteen percent of patients with infected diabetic foot ulcers (DFUs) eventually require lower limb amputations to prevent life‐threatening sepsis.^[^ ^2^ ^]^ Prompt and convenient wound cleansing options, along with innovative non‐antibiotic therapies that can be repeatedly administered at home, offer an effective alternative for managing diabetic and other chronic wound infections.

Current anti‐biofilm research focuses on direct killing of the dormant bacteria embedded in biofilms, known as persisters. Persisters can evade antibiotic attacks because conventional bactericides primarily target actively growing cells.^[^ ^3^ ^]^ Membrane‐attacking agents, such as colistin and other polycationic molecules, despite their nephrotoxicity, appear to be the last resort against persisters, as they can directly rupture bacteria regardless of their metabolic state.^[^ ^4^ ^]^ Other ATP‐independent agents like acyldepsipeptide and lassomycin are emerging but remain inactive against Gram‐negative persister cells.^[^ ^5^ ^]^

In addition to direct killing, approaches targeting bacterial extracellular matrices are gaining attention. Bacteria themselves account for only 5–35% of the biofilm volume, with the remaining portion occupied by extracellular polymeric substances (EPS) and water.^[^ ^6^ ^]^ EPS is primarily composed of polysaccharides, extracellular DNA (eDNA), and proteins. Correspondingly, enzymatic degradation methods using glycosidase, DNase, and proteinase have been developed to specifically disperse biofilms, which unmasks the bacteria for enhanced neutrophil‐mediated killing^[^ ^7^ ^]^ and the reversal of antibiotic tolerance.^[^ ^8^ ^]^ An example is the DispersinB wound gel developed by Kane Biotech Inc., which is currently under clinical trials.^[^ ^9^ ^]^ However, the EPS component targeted by a dispersing enzyme may not be universally present across various biofilms and thus may be effective only in some biofilms. Research on dispersing enzymes has mostly focused on mono‐species biofilms, overlooking the polymicrobial biofilms prevalent in nature, potentially oversimplifying the complexity of chronic wound infections. Additionally, dispersing enzymes are not inherently bactericidal, underscoring their need for co‐administration with antibiotics.^[^ ^10^ ^]^ Even though it is painful and requires anesthesia, mechanical methods like sharp debridement and rigorous wiping with gauze continue to be the most effective strategies for combating wound biofilms. Bacteria, once released from disrupted biofilms and left unprotected, can be effectively eliminated through saline cleansing or by host immune clearance, such as phagocytosis. Thus, the importance of sufficient disruption of biofilms, whether the biofilms are mono‐species or polymicrobial, cannot be overstated.

Blue light (BL) in the range of 400–470 nm represents an emerging antimicrobial modality that targets both planktonic and biofilm‐encased cells.^[^ ^11^ ^]^ It is widely accepted that blue light kills bacteria by exciting endogenous photosensitizers such as porphyrins within bacterial cells, generating detrimental Reactive Oxygen Species (ROS) that damage the pathogens. Recent discoveries have unveiled a growing list of bacterial intracellular targets responsive to blue light, including pyocyanin, staphyloxanthin, and catalase.^[^ ^12^ ^]^ Despite these advances, direct evidence of blue light affecting the EPS components of biofilms remains limited. Among the few examples, Martegani et al. observed that 455‐nm blue light could profoundly affect the biofilm matrix without compromising the viability of *Pseudomonas aeruginosa* PA01 cells.^[^ ^13^ ^]^ A combination of blue light with bioactive compounds, such as quinine, carvacrol, or thymol, significantly impacted the integrity of biofilms as well.^[^ ^14^ ^]^ However, it remains elusive whether the light‐based treatments directly disrupt the biofilm matrix, or the bactericidal activity of the treatment indirectly affects the overall structure of the biofilm. Moreover, these combinations appeared to be ineffective against biofilms formed by blue‐light refractory bacteria, such as *K. pneumoniae* and *E. coli*. These BL‐refractory bacteria frequently co‐infect chronic wounds with BL‐susceptible ones, forming multi‐species biofilms (Graphic summary: Left). While blue light alone or alongside compounds like carvacrol, kills BL‐susceptible bacteria, the refractory ones resurge, leaving the biofilm more difficult to treat (Graphic Summary: upper right). These co‐infected, BL‐refractory bacteria produce limited amounts of porphyrins.^[^ ^15^ ^]^ Apparently, alternative approaches capable of killing both BL‐susceptible and BL‐refractory pathogens, independent of porphyrin production, can be sufficient complements to effectively treat polymicrobial biofilms by light‐based therapy.

In this study, we delved into a non‐canonical mechanism through which blue light (BL), particularly at 405 nm, disassembles biofilm EPS in a porphyrin‐independent fashion. Fe(III) was serendipitously found in our study to degrade EPS polysaccharides and eradicate embedded pathogens in biofilms upon BL exposure (Graphic Summary: lower right). Moreover, the photolytic effect of the BL‐Fe(III) treatment on the biofilm matrix synergized with host immune clearance and bactericidal antibiotics to combat polymicrobial biofilm wound infections. This innovative photolysis of biofilms extends to both BL‐susceptible and ‐refractory pathogens as well as Gram‐positive and Gram‐negative pathogens, greatly broadening the spectrum of BL‐mediated bactericidal activity. The BL‐Fe(III) regimen holds great promise as a safe, efficient, and painless wound‐cleansing modality, and can potentially revolutionize the care for patients with chronic wounds and conditions such as bedsores, diabetic ulcers, and necrotizing fasciitis.

## 2 Results

### 2.1 Complete Mucoidal Disruption of the Hypermucoid *K. pneumoniae* by 405‐nm Blue Light (BL)

**Figure 1:**
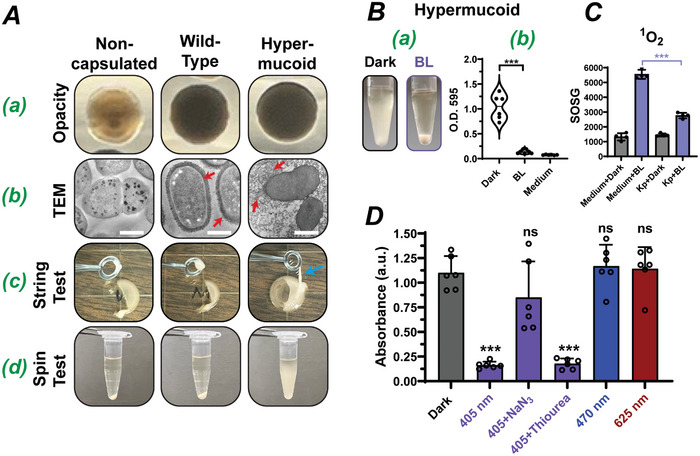
Exposing hypermucoid *K. pneumoniae* to 405‐nm BL leads to a decrease in the culture viscosity. A) Colony morphologies of different *K. pneumoniae* mutants. a) Translucent (non‐capsulated mutant) and opaque [wild‐type (WT) and hypermucoid mutant] colonies with similar sizes. b) Transmission electron microscopy (TEM) images. Red arrows in WT and hypermucoid strains indicated capsule polymers. c) String test. Neither the non‐capsulated mutant nor the WT colony was slimy. A blue arrow, a string drawn from hypermucoid colony. d) Spin test. The non‐capsulated and WT liquid cultures, but not the hypermucoid bacteria, formed pellets after centrifugation. B) Exposure to 405‐nm blue light led to the sedimentation of hypermucoid culture. a) Spin test of hypermucoid culture with or without BL treatment. b) Absorbance measurements (O.D._595_) of the upper culture layer post‐centrifugation. C) Singlet oxygen (^1^O_2_) production in BHI medium or hypermucoid culture upon blue light irradiation. D) Changes of viscosity in the hypermucoid culture by different wavelengths of lights and antioxidants. NaN_3_, 10 mM; Thiourea,150 µM. Power intensity of BL, 80 J cm^−2^ in A, B, and D; and 10 J cm^−2^ in C. n = 4 in C; and 6 in B and D; Data are presented as means ± SD. ***p ≤ 0.001 compared in the presence and absence of BL. ns, no significance. Scale bar in A (b), 500 nm.

*Klebsiella (K). pneumoniae*, a BL‐refractory bacterial strain, can undergo rapid phenotypic switches to adapt various host niches.^[^ ^16^ ^]^ For instance, a wild‐type bacterium can spontaneously switch to either a non‐capsular or hyper‐capsular (hypermucoid) phenotype by mutations at the capsule biosynthesis locus^[^ ^16a^ ^]^ (**Figure** **1**A). The non‐capsular mutant is better adapted for invasion and persistence in epithelial cells during urinary tract infections. Meanwhile, the hyper‐mucoid mutant is associated with bloodstream dissemination, as the abundant capsule polysaccharide confers resistance to phagocytosis. On the other hand, wild‐type (WT) strains are found across various infection sites, including wounds, abscesses, and lungs, where the bacteria can further evolve to adopt different infection strategies—namely, to persist locally or spread systemically.^[^ ^16a^ ^]^

The non‐capsulated mutant could spontaneously develop when the WT was cultured on agar for approximately three days; in contrast, the hypermucoid mutant was obtained by a culturing condition that promoted biofilm growth over two days (Figure S1, Supporting Information and Experimental Section). The hypermucoid *K. pneumoniae* served as an excellent model for our initial investigation of BL in biofilm disruption because this bacterium is BL‐refractory (Figure S2, Supporting Information) and produces abundant EPS material (i.e., the capsule polymers). Accordingly, we isolated these spontaneous mutants and characterized their unique colony morphologies: the non‐capsular mutant was translucent, whereas the WT and hypermucoid mutants were opaque (Figure 1Aa, opacity). The opaque phenotype may be attributed to capsular materials, increasing the colony's refractive index. The distinct colony phenotypes were verified by transmission electron microscopy (Figure 1Ab, TEM). The non‐capsulated mutant showed no visible extracellular substance whereas the WT bacteria were each surrounded by a thin layer of capsular polysaccharide (red arrows), insufficient for cell bridging. In marked contrast, the hypermucoid mutants produced extensive capsular filaments that entangled and occupied the extracellular space between bacteria, holding the cells together. In agreement with the TEM images, the non‐capsular and WT *K. pneumoniae* grown on agar were easily scraped off by an inoculation loop. Conversely, the hypermucoid colony was rooted in the agar and difficult to disrupt by the same loop. Additionally, the hypermucoid colony was sticky, and a string of cells (>5 mm) could be easily drawn out by a loop (blue arrow), and as such it was also called a string test (Figure 1Ac). There was no such a string in the non‐capsular and WT colonies under similar conditions. If cultured overnight in liquid media, both the non‐capsular and WT cultures could be easily spun down, evidenced with clear supernatant and a pellet in the tubes (Figure 1Ad). In contrast, the hypermucoid mutant could not be pelleted by centrifugation due to the viscosity of its culture. This viscous phenotype suggests the presence of a dense network in the culture, where cells are entangled or embedded. Thus, the hypermucoid culture and the spin test^[^ ^17^ ^]^ were used to study the potential effect of blue light on the extracellular matrix. Surprisingly, the hypermucoid culture was successfully spun down by centrifugation after blue light irradiation at 405 nm for 80 J cm^−2^ (Figure 1B). In fact, the absorbance of the supernatant after centrifugation was similar to the absorbance of the medium control (Figure 1Bb), arguing for nearly complete disassembly of the hyper‐mucoidal network (Figure 1Ba). Based on the decrease in optical density, it was estimated that 93.5% of the bacteria were sedimented in the presence versus absence of BL irradiation (Figure 1Bb). Blue light irradiation is often associated with surged production of singlet oxygen (^1^O_2_) or reactive oxygen species (ROS) as a result of photodynamic reactions,^[^ ^11^ ^]^ which can cause mucoidal disruption. To our surprise, ^1^O_2_ was generated at the highest level in the BHI medium alone upon BL irradiation, suggesting the presence of BL‐responsive “photosensitizers” in the medium, not in the bacteria (Figure 1C). It is thus imperative for us to identify the BL‐responsive compound(s) in the medium if we were to apply this modality to disrupt the biofilm in the wounds. The ^1^O_2_ levels seemed significantly attenuated in the hypermucoid culture, which was probably ascribed to the consumption or absorbance of the ^1^O_2_ by the mucoid materials. Nevertheless, the importance of BL in mucoidal disruption was corroborated since only 405‐nm blue light, rather than other wavelengths (470 nm and 625 nm), could result in sedimentation of the hypermucoid bacteria in spin tests (Figure 1D). The BL‐mediated sedimentation could be partially reversed by a NaN_3_, a ^1^O_2_ scavenger, but not by thiourea, a hydroxyl radical (OH·) scavenger, ruling out the involvement of a conventional type I photodynamic reaction in the disruption of the hypermucoid bacteria.

### 2.2 BL Combined Fe(III) Degrades Capsule Polysaccharide and Potentiates Phagocytosis of *K. pneumoniae*

**Figure 2:**
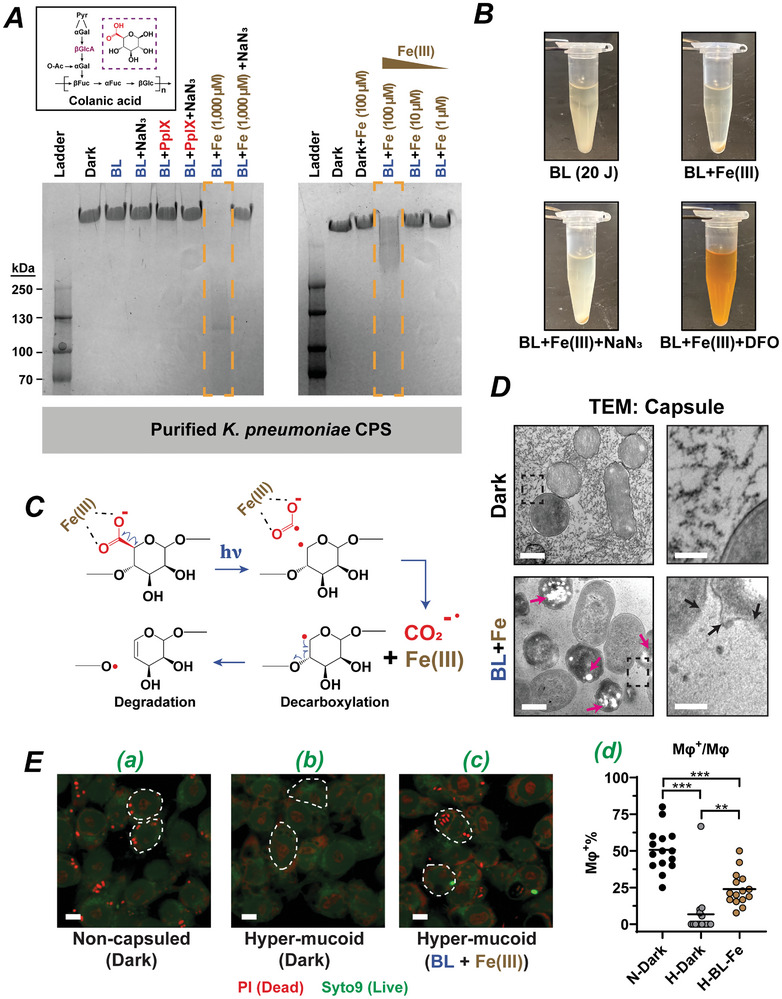
BL‐Fe(III) catalyzes the breakdown of capsular polysaccharides in *K. pneumoniae*. A) Alcian blue staining of the treated capsular polysaccharides (10 µg per Lane) purified from hypermucoid *K. pneumoniae*. Yellow dashed boxes highlighted the degradation of capsule material. BL, 405‐nm blue light (80 J cm^−2^). PpIX, protoporphyrin IX (10 µM). NaN_3_, sodium azide (10 mM). The black solid box showed the chemical structure of colanic acid, a common type of capsular polymer produced by *K. pneumoniae*. Magenta dashed box highlighted the D‐glucuronic acid (βGlcA), containing a carboxyl group. B) The addition of Fe(III) to the hypermucoid culture increases its susceptibility to blue light. Fe (III), 300 µM. BL, 20 J cm^−2^. This degradation was blocked by ferric neutralizing agents, NaN_3_ or deferoxamine (DFO). C) A depict of the mechanism for the breakdown of capsular polysaccharides by blue light and Fe(III). Fe(III)‐coordination catalyzes the photolysis of carboxylate, generating free radicals and leading to subsequent breakdown of the polymer chain. D) TEM images validated the breakdown of the capsular polysaccharides after BL‐Fe(III) treatment. The area in black dashed box on the left is enlarged in the corresponding right panels, showing fragmented capsule filaments after BL‐Fe(III) treatment. Magenta arrows indicate damaged cells after treatment. Black arrow indicates ruptured bacterial membranes. E) Macrophage‐mediated phagocytosis of a) non‐capsular mutant, b) hypermucoid mutant, and c) BL and Fe(III) treated hypermucoid mutant. PI, red, dead bacteria. Syto9, green, live bacteria. Dashed areas outlined some representative macrophage cells. d) Percentage of activated macrophage cells in each group. Mφ^+^, an activated macrophage that has engulfed at least one bacterium. The Mφ^+^/Mφ ratio was determined from 15 randomly selected fields across three independent experiments. **p ≤ 0.01. ***p ≤ 0.001 compared between indicated groups. Scale bars, 500 nm in D, left, 125 nm in D, right, and 5 µm in E.

To decipher whether a Type II photodynamic reaction was the underlying mechanism, it was necessary to irradiate the capsular materials from the hypermucoid culture with BL in the presence of type II photosensitizers in saline solution in place of BHI medium. The capsular polysaccharides of Gram‐negative and Gram‐positive bacteria exhibit significant biochemical and structural variety. Despite their diversity, capsular polysaccharides are largely acidic and negatively charged, owing to the presence of sugar components that contain carboxylic acid.^[^ ^18^ ^]^ *K. pneumoniae* strains can produce over 80 capsule serotypes. Inset in **Figure** **2**A illustrates the structure of colanic acid, a classic capsular polysaccharide that contains one carboxylic acid. We purified the capsule material from the hypermucoid culture of *K. pneumoniae* (*Kp*) and treated it with BL alone or BL plus NaN_3_. Both treatments failed to disrupt the hypermucoid structure, as manifested by the intact bands of high molecular weight, comparable to those in the dark control (Figure 2A). This confirmed the presence of the “photosensitizer” in the medium, not in the bacteria. We then added protoporphyrin IX (PpIX) to the capsular polymer, followed by BL irraditation. PpIX is a representative of bacterial porphyrin, functions as a type II photosensitizer, and generates ^1^O_2_ upon BL irradiation. To our surprise, the capsular polymer was again resistant to BL combined with PpIX, even with a high concentration of PpIX (10 µM), which was over 100‐fold higher than that in *E. coli* cells (Figure 2A).^[^ ^15^ ^]^ These observations raised an intriguing possibility that a completely novel mechanism was involved in the observed disruption of the hypermucoid structure by BL (Figure 1).

A recent study demonstrated that BL and Fe(III) could degrade carboxylate‐containing polysaccharides.^[^ ^19^ ^]^ As depicted in Figure 2C, Fe(III) and the carboxylate form coordination complex, presenting a ligand‐to‐metal charge transfer (LMCT) absorption band at 405‐nm blue light. Blue light irradiation lyses the carboxylate bond, leading to a sugar radical and a free CO_2_^•^−, which is stabilized by nearby iron. Translocation of the lone electron leads to the cleavage of the glycosidic bond and subsequent degradation of the polysaccharide (Figure 2C). Inspired by this photoreaction, we evaluated the potential of BL and Fe(III) photolysis of the capsular polysaccharides containing carboxylic groups. The combination of BL and Fe(III) (1000 µM) could rapidly break down the capsular polymer, as evidenced by the complete disappearance of the band (Figure 2A). The degradation of capsule by Fe(III) was dose‐dependent: at 1000 µM, there was complete degradation; at 100 µM, the degradation was partial, shown by higher molecular weight smears. Below 10 µM, Fe(III) had a minimal degradation effect. Interestingly, NaN_3_ could abrogate the photolytic effect of BL‐Fe(III), but it did not function as a ^1^O_2_ scavenger, as it typically did in canonical photoreactions (Figure 2A). Rather NaN_3_ and Fe(III) formed an iron azide (FeN_3_) precipitation, blocking the photolysis reaction by sequestering iron. To further verify the effect of Fe(III), we repeated the mucoidal disruption experiment with a lower blue light energy (i.e., 20 J cm^−2^), which was only 25% of the original, unable to cause overt sedimentation (Figure 2B, top left). Addition of Fe(III) at 300 µM efficiently reduced the culture viscosity and resulted in sedimentation after blue light irradiation (top right). Consistent with previous observation, NaN_3_ and Fe(III) formed precipitation and blocked photolysis (bottom left). Most importantly, adding deferoxamine (DFO), a strong and specific chelator of ferric ion, also effectively inhibited the photolysis and sedimentation, suggesting the essential role of Fe(III) in the mucoidal disruption triggered by blue light (bottom right).

The degradation was also confirmed by transmission electron microscopy, revealing breakdown of the extracellular filaments as indicated by the clear background between bacterial cells following the treatment (Figure 2D, bottom left). Occasionally, small dots (<50 µm) were observed in the extracellular space, possibly representing fragments of larger polymers after photolysis (Figure 2D, bottom right). In contrast, the capsular polymers in the dark control group were more evident, tightly knotted, and occupied the extracellular space fully (Figure 2D, top). Beyond affecting the capsule structure, the BL‐Fe(III) treatment also seemed to damage the bacterial membranes, leading to leakage of cell contents (Figure 2D, bottom). The magenta arrows indicated loss of cell contents, while the black arrows showed ruptured membranes. The hypermucoid capsule functions as a safeguard against phagocytosis and its disruption could enhance phagocytosis. This is because the copious capsule can mask the pathogen‐associated molecular patterns (PAMPs), such as lipopolysaccharides (LPS), on the bacterial surface that normally trigger phagocytosis.^[^ ^16^, ^17^, ^20^ ^]^ In agreement with this, the capsule‐deficient mutants were found to be more efficiently internalized and killed by mouse macrophages (RAW 264.7) (Figure 2Ea). Conversely, the hypermucoid mutant was immune to both attachment and phagocytosis, with few bacteria seen within the macrophages (Figure 2Eb). As expected, the phagocytosis resistance of the hypermucoid mutant was partially but significantly reversed following treatment with BL and Fe(III) (Figure 2Ec). The percentages of activated macrophages (Mφ+/Mφ) that had engulfed bacteria after being co‐cultured with different *K. pneumoniae* cells for 2 hours were shown in Figure 2Ed. The non‐capsulated strain triggered 50.7% of Mφ activation, while the hyper‐mucoid mutant activated only 6.7% of Mφ, which was significantly increased to 23.9% after BL and Fe(III) treatment, albeit at lesser degree than the phagocytosis of the non‐capsulated mutant.

### 2.3 BL‐Fe(III) Decomposes the Alginate Matrix Produced by *P. aeruginosa*

**Figure 3:**
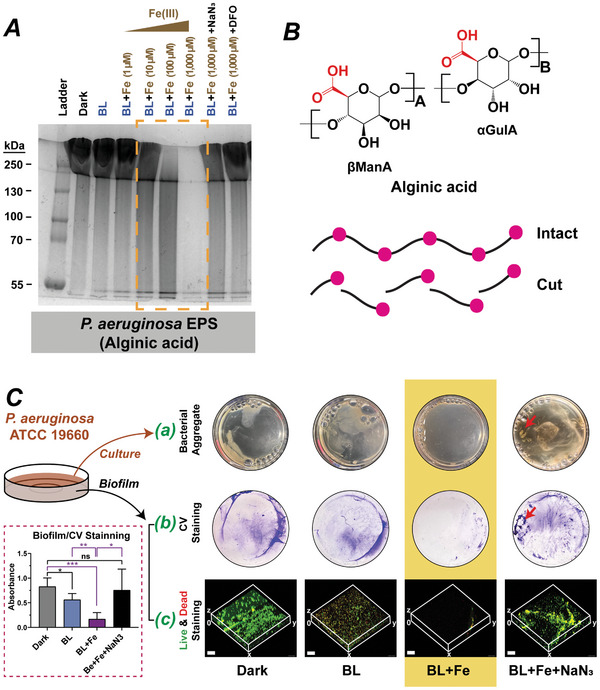
BL‐Fe(III) breaks down extracellular polysaccharides in *P. aeruginosa*. A) Alcian blue staining of treated alginic acid (dissolved in saline, 25 µg per Lane). Yellow dashed box highlighted the degraded alginic acid. BL, 405‐nm blue light at 80 J cm^−2^; NaN_3_, sodium azide at 10 mM; and DFO, deferoxamine, a Fe(III) chelator, at 7.5 mM. B) Chemical structure of alginic acid and an illustration of the breakdown. *βManA*, β‐D‐mannuronic acids and *αGulA*, α‐L‐guluronic acids. C) BL and Fe(III) compromised the structural integrity of *P. aeruginosa* aggregate and biofilm. a) Bacterial aggregates. b) Crystal violet staining of the treated biofilms. Magenta dashed box, quantification of the remaining biomass stained by crystal violet (n = 4). Data are presented as means ± SD. *p ≤ 0.05, **p ≤ 0.01, ***p ≤ 0.001, and ns, no significant difference between indicated groups. BL, 40 J cm^−2^; Fe(III), 300 µM; and red arrows, FeN_3_ precipitations. c) Confocal microscopy of the biofilm viability and integrity after treatments. Scale bar, 50 µm.

To extend the matrix degradation property of BL‐Fe(III) regimen to other bacteria, ***Pseudomonas*** *(P). aeruginosa* (*Pa*) was investigated. *Pa* expresses at least three types of exopolysaccharides (Psl, Pel, and alginate), all of which are involved in biofilm growth.^[^ ^21^ ^]^ Psl and Pel are neutral and cationic polysaccharides, respectively, and are primarily synthesized by non‐mucoid strains such as PA01 and PA14 to form surface‐attached biofilms. Mucoid strains such as *P. aeruginosa* 19 660 are commonly isolated from cystic fibrosis or sepsis patients and overproduce alginate. Alginate (**Figure** **3**B) is a carboxyl‐containing polymer that connects different cells to form thicker 3D biofilms.^[^ ^22^ ^]^ Similar to our observation with *K. pneumoniae* capsule, BL and Fe(III) could degrade the alginate polymer in a dose‐dependent manner. This degradation could be inhibited by NaN_3_ or DFO, the Fe(III) chelator (Figure 3A). We also compared the degradation efficiency between Fe(III) and Fe(II): Fe(III) showed much better degradation than Fe(II) under blue light irradiation (Figure S3, Supporting Information). Fe(II) showed some degree of degradation, likely due to blue light oxidizing some Fe(II) to Fe(III). Additionally, we confirmed that the BL‐Fe(III) treatment selectively targets carboxyl‐containing sugars, as evidenced by its inability to degrade starch, a polymer of glucose subunits (Figure S4, Supporting Information). This suggests that the BL‐Fe(III) modality is specific in its action; the breakdown of the EPS polymer is not attributed to the generation of non‐selective reactive oxygen species (ROS), such as hydroxyl radicals (•HO).

After growing *P. aeruginosa* 19 660 statically for two days, a mucoid and viscous culture was obtained. To test the degradation effect on the biofilm, we divided the culture into two parts. The first part was the liquid culture containing the bacterial aggregate, which was strong enough to resist mechanical forces such as pipetting up and down. The second part consisted of the loosely‐attached, sheet‐like biofilms. Both parts were treated as follows: Dark, BL alone, BL‐Fe(III), and BL‐Fe(III) supplemented with NaN_3_. After the BL‐Fe(III) treatment: i) the bacterial aggregate could be easily dispersed by mechanical forces (Figure 3Ca), and ii) The attached biofilms became significantly weaker and were readily dislodged during the washing steps in crystal violet staining (Figure 3Cb and magenta dashed box). In marked contrast, the BL‐Fe(III) supplemented with NaN_3_ did not alter integrity in bacterial aggregate or biofilms. BL alone exhibited some antibiofilm effects as evidenced by the reduced biomass stained by crystal violet. This was likely attributed to the collapse of the cells embedded within the biofilm, as *P. aeruginosa* was known to be highly sensitive to blue light inactivation. Therefore, we reduced the blue light fluence to 40 J cm^−^^2^ in this experiment. Confocal microscopy recapitulated the results from crystal violet staining, reaffirming that the biofilm was largely dispersed after the BL‐Fe(III) treatment (Figure 3Cc, BL+Fe group). In addition, the fluorescence images revealed a mixed population (in yellow color) of live and dead bacteria in the biofilm after blue light (BL) irradiation (Figure 3Cc, BL alone group), indicating that blue light alone could not eradicate the biofilms‐embedded P. aeruginosa. Notably, adding NaN_3_ could inhibit the activity of BL and Fe(III), resulting in some visible FeN_3_ precipitates (Figure 3Cab, red arrows).

### 2.4 BL‐Fe(III) Disrupts Polymicrobial Biofilms and Enhances the Efficiency of Ciprofloxacin

We have demonstrated that BL‐Fe(III) degrades exopolysaccharides in two mono‐species biofilms—the mucoid *K. pneumoniae* (*Kp*) and *P. aeruginosa* (*Pa*). Diabetic wound infections often involve a polymicrobial community, with the most frequently isolated bacteria from diabetic foot samples being *S. aureus*, *P. aeruginosa*, *K. pneumoniae*, and *A. baumannii*.^[^ ^23^ ^]^ To investigate the broad applications of BL‐Fe(III), we employed a previously established polymicrobial biofilm model.^[^ ^24^ ^]^ This multi‐species biofilm comprised *P. aeruginosa* (≈80%), *K. pneumoniae* (≈10%), *A. baumannii* (6%), and *S. aureus* (3%), all grown in a wound‐like environment containing plasma, erythrocytes, and a chopped‐meat‐based medium. Polymicrobial biofilms were grown on purpose to encompass BL‐recalcitrant (i.e., *K. pneumoniae*) and BL‐sensitive (*S. aureus*, *P. aeruginosa*, and *A. baumannii*) bacteria and both Gram‐positive and Gram‐negative multi‐drug resistant strains. To avoid an overabundance of *Kp* capsule and *Pa* alginic acids in the biofilm matrix, which were extensively studied earlier, we specifically chose the wild‐type strains of *Pa* PAO1 and *Kp* IQ0035 instead of mucoid strains for the inoculum. This approach was adopted to ensure that the polymicrobial biofilm matrix more accurately represents a diverse range of polymers.

**Figure 4:**
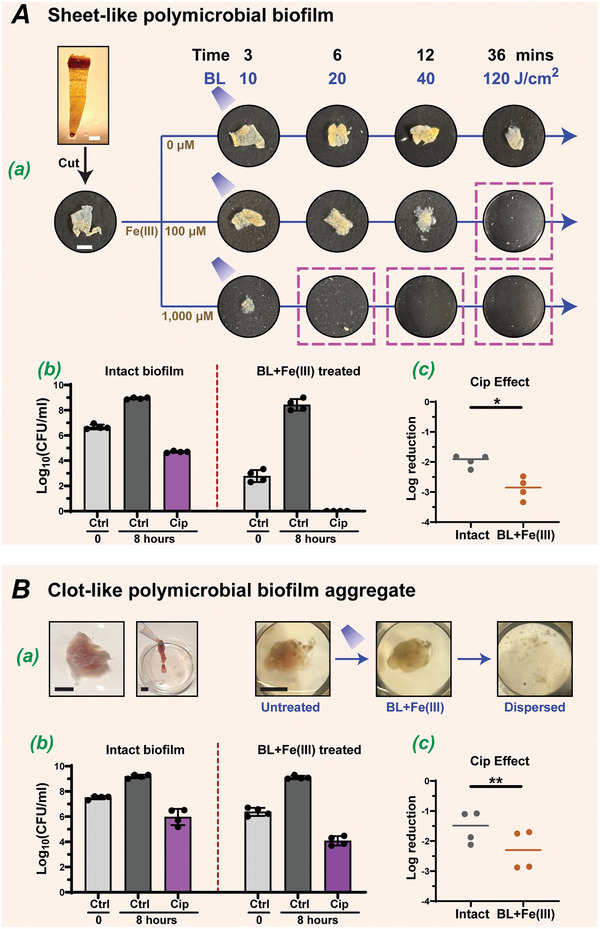
BL‐Fe(III) disrupts polymicrobial biofilms and potentiates the bactericidal activity of ciprofloxacin. Both sheet‐like A) and clot‐like B) polymicrobial biofilms consisted of four common wound bacteria, including *P. aeruginosa*, *K. pneumoniae*, *S. aureus*, and *A. baumannii*. (A). BL‐Fe(III) dose‐dependent disruption of the integrity of the sheet‐like biofilm (a). Magenta dashed boxes highlighted the combinations of BL and Fe(III) where the sheet‐like biofilms were disrupted after treatment. (B). Photos of the clot‐like polymicrobial biofilm aggregates (a, left) and BL‐Fe(III) breaking down the clot‐like biofilm (a, right). Overall survivals (Ab and Bb) and Log reductions (Ac and Bc) of the intact or BL‐Fe(III) treated biofilms after ciprofloxacin (Cip, 10 µg mL^−1^) incubation. Scale bars, 3 mm. BL and Fe(III) in Ab and B were set at 40 J cm^−2^ and 300 µM, respectively. n = 4. Data are presented as means ± SD. *p ≤ 0.05, **p ≤ 0.01 compared between indicated groups.

This mixture of strains grown in the same wound‐like medium could result in two different forms of polymicrobial biofilms: a sheet‐like biofilm (**Figure** **4**A) when grown under shaking conditions, and a clot‐like biofilm aggregate (Figure 4B) when not shaken. The coagulase‐positive *S. aureus* IQ0064 promoted plasma coagulation with the clotting effect encouraged under the non‐shaken condition, providing a 3D scaffold that accommodated multiple bacterial species. Under shaking conditions, the shear force mechanically disrupted coagulation by preventing the necessary interactions between fibrin monomers or hindering the formation of a stable scaffold for fibrin accumulation. As a result, mixed bacteria tended to attach to the provided surface (e.g., a pipette tip) and connected primarily with other bacteria/species through the extracellular matrix. The sheet‐like biofilm formed was flat and could be peeled off the pipette tip surface, while the clot‐like biofilm was bulkier and resembled a hydrogel structure. In our observations, the biofilm found in mouse wounds appeared more sheet‐like, possibly due to the thin skin and limited bleeding in mouse wounds, resulting in less plasma. However, this sheet‐like biofilm in mouse wounds was too thin to be seen by the naked eye and was further complicated by extensive neutrophil infiltration. In contrast, the morphology and anatomy of the clot‐like biofilm are comparable to those found in human chronic wounds, which typically have deeper wound beds and more bleeding.^[^ ^25^ ^]^

The sheet‐like biofilm was cut into small pieces and then treated with blue light and various concentrations of Fe(III): 0, 100, and 1000 µM (Figure 4Aa). Blue light alone, even at the maximum power density tested (120 J cm^−^^2^), was unable to affect the structural integrity of the biofilm. In marked contrast, the addition of Fe(III) rapidly degraded the matrix when exposed to blue light irradiation. The degradation effects became increasingly prominent as an increase in the total energy of the blue light and the concentration of Fe(III). Remarkably, the polymicrobial biofilm was almost completely dispersed by minimal mechanical forces (such as pipetting up and down several times) after 6 minutes of irradiation when Fe(III) was used at 1000 µM. Video clips revealed that polymicrobial biofilms, which were usually resilient, became vulnerable to mild physical disruptions following a brief BL‐Fe(III) treatment (Figure S5, Supporting Information). The BL‐Fe(III) at 40 J cm^−2^ and 300 µM also compromised the clot‐like biofilm aggregate, making it more susceptible to physical dispersion (Figure 4Ba).

Bactericidal antibiotics only kill actively growing bacteria. This is why drugs like Ciprofloxacin (Cip) are more effective against bacteria in the logarithmic phase than those in the stationary phase (Figure S6, Supporting Information). Similarly, biofilm‐embedded bacteria are usually more tolerant to bactericidal drugs because these bacterial aggregates have a lower surface‐to‐volume ratio, are less exposed to nutrients, and therefore have a slower metabolism, which helps them withstand high dosages of antibiotics. We hypothesized that using BL‐Fe(III) to disperse the polymicrobial biofilm would increase the surface‐to‐volume ratio of the bacterial population, encourage their regrowth in BHI medium, and partially reverse drug tolerance. To test this, biofilm samples—intact or dispersed by BL‐Fe(III)—were allowed recovered in fresh BHI medium with or without Cip for 8 hours (Figure 4Ab,Bb). In the absence of Cip, biofilm bacteria rapidly proliferated in the medium, reaching a high number of 9 Log^CFU^ by the 8th hour across all groups. For the sheet‐like biofilm (Figure 4Ab), Cip alone (10 µg mL^−1^, ≥10×MIC) or BL‐Fe(III) alone (40 J cm^−^^2^ and 300 µM) killed 1.97 and 3.88 Log^CFU^ of biofilm bacteria, respectively. Yet, combining BL‐Fe(III) and Cip could eliminate all bacteria, resulting in a 6.66 Log^CFU^ reduction. The bactericidal effect of Cip on the BL‐Fe(III) dispersed sheet‐like biofilm was measured at ‐2.88 Log^CFU^, which was significantly better than its effect (‐1.97 Log^CFU^) on intact biofilm (Figure 4Ac). For the clot‐like biofilm aggregate, all of the above‐mentioned effects were attenuated, probably because the thickness of the biofilm hindered the penetration of light, Fe(III), and the Cip antibiotic. Despite such attenuation, the combination of BL‐Fe(III) and Cip synergistically killed 3.43 Log^CFU^ of biofilm‐embedded bacteria, representative of more than 99.9% reduction (Figure 4Bb). The Cip effect was improved from a ‐1.54 Log^CFU^ on intact biofilm to a ‐2.2 Log^CFU^ on the dispersed biofilm (Figure 4Bc).

### 2.5 BL‐Fe(III) Exhibits a Broad Spectrum of Antimicrobial Activity but Spares Host Skin Fibroblasts

**Figure 5:**
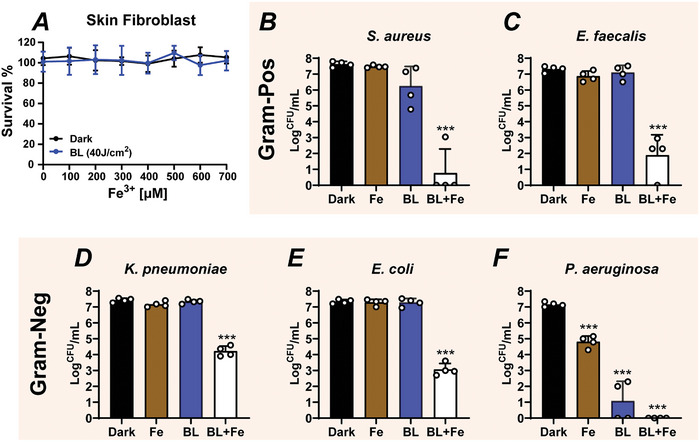
BL‐Fe(III) kills a panel of bacteria while sparing host skin fibroblasts. A) BL exposure and increasing concentrations of Fe(III) up to 700 µM did not affect the viability of human skin fibroblasts. BL, 40 J cm^−2^. n = 3. B‐F) Antimicrobial activity of the BL‐Fe(III) modality. Bacterial cultures were grown to stationary phase and diluted to O.D._595_ of 0.05, ≈1‐5×10^7^ CFU mL^−1^. The cultures were exposed to BL‐Fe(III) with BL at 40 J/cm^2^ and Fe(III) at 300 µM. n = 4. Data are presented as means ± SD. ***p ≤ 0.001 compared between the Dark and the BL‐Fe(III) groups.

A brief application of ferric solution, for instance, Monsel's solution containing 20% ferric sulfate or ≈1 M Fe(III)), to soft tissue is commonly used as a hemostatic agent in dermatology and dentistry.^[^ ^26^ ^]^ Due to the formation of a coagulum, systemic absorption of Fe(III) is minimal. Other sources also indicate limited evidence of the carcinogenicity or toxicity of Fe ions (e.g., at doses of 2g kg^−1^) through dermal exposure, inhalation, and oral intake.^[^ ^27^ ^]^ In this study, we explored the potential of BL‐Fe(III) as a wound‐cleansing method, which also involved short‐term exposure to Fe(III). To evaluate its safety, we treated human skin fibroblasts with BL at 40 J cm^−^^2^ for 12 min and increasing concentrations of Fe(III) up to 700 µM and did not find any signs of cytotoxicity (**Figure** **5**A). We then proceeded to evaluate the antimicrobial effectiveness of BL‐Fe(III) treatment against a panel of pathogens (Figure 5B‐F). The panel included a BL‐sensitive strain of *P. aeruginosa* (*Pa*), an intermediately sensitive *S. aureus* (*Sa*), and three BL‐tolerant bacteria: *E. coli* (*Ec*), *K. pneumoniae* (*Kp*), and *E. faecalis* (*Ef*). This selection covers two Gram‐positive strains, namely *Sa* and *Ef*, and three Gram‐negative strains, specifically *Pa*, *Ec*, and *Kp*. Surprisingly, the BL‐Fe(III) combination was effective against all bacterial strains tested, as evident by >3‐Log reduction in viable CFU counts (>99.9% inactivation), which was a stringent yet widely recognized criterion for determining bactericidal activity. Also, the BL‐Fe(III) treatment killed the BL‐sensitive strains (*Pa*, 7.1‐Log; *Sa*, 6.8‐Log CFU reduction) more efficiently than it did on BL‐recalcitrant strains (*Kp*, 3.2‐Log; *Ec*, 4.2‐Log; *Ef*, 5.4‐Log). Notably, *E. faecalis* (*Ef*) does not produce porphyrins and shows high tolerance to BL‐mediated killing. The effective killing of the enlisted bacteria by BL‐Fe(III) suggests that its bactericidal activity involves endogenous porphyrins‐dependent and independent mechanism. While the porphyrins‐dependent mechanism has been extensively explored, porphyrins‐independent mechanism is completely novel. It may target vital carboxyl‐containing components on the bacterial surface. The dual antimicrobial mechanisms of BL‐Fe(III) regimen warrant the broad bactericidal spectrum, crucial for managing multi‐species biofilms.

**Figure 6:**
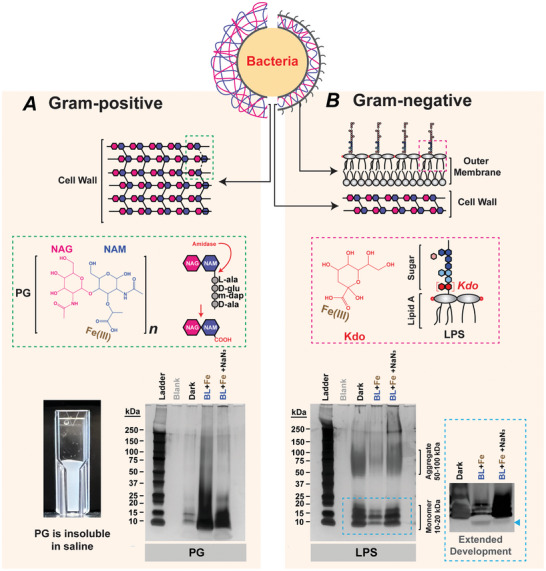
BL‐Fe(III) degrades bacterial surface components with repeated carboxyl groups. A) Silver staining of treated peptidoglycan (PG, 5 µg per Lane). NAM, N‐acetylmuramic acid. NAG, N‐acetylglucosamine. The green dashed box highlighted amidase removing the oligopeptide crosslinker, exposing a free carboxyl group in the bacterial cell wall. B) Silver staining of treated lipopolysaccharide (LPS, 5 µg per Lane). Kdo, Ketodeoxyoctonic acid. The magenta dashed box illustrated the location of Kdo in an LPS molecule. The cyan dashed box highlighted the fainter bands of LPS monomer at 10‐20 kDa. The blue triangle indicated an emerged LPS fragment below 10 kDa after BL and Fe(III) treatment (with extended gel development during silver staining). BL, 80 J cm^−2^. Fe(III), 300 µM.

The broad‐spectrum antimicrobial activity of BL‐Fe(III) in planktonic cultures suggests that it may also directly target bacterial surface components apart from the extracellular matrix, in agreement with TEM observations (Figure 2D). In this regard, peptidoglycan (PG) is the outermost cell wall layer in Gram‐positive pathogens, while lipopolysaccharides (LPS) are located on the outer leaflet of the outer membrane of Gram‐negative pathogens (**Figure** **6**A,B, top). PG is composed of repeating units of N‐acetylglucosamine (NAG) and N‐acetylmuramic acid (NAM). Notably, NAM contains a carboxyl group (Figure 6A, green dashed box), which is typically masked by crosslinking oligopeptides. However, this carboxyl group becomes exposed due to the action of amidase, as the cell wall undergoes dynamic remodeling to accommodate cell growth and division. PG is insoluble (Figure 6A, cuvette); therefore, the high‐molecular‐weight polymer remained invisible following gel electrophoresis (Figure 6A, gel image). Intriguingly, after treatment with BL‐Fe(III), there was a substantial increase in the more soluble low‐molecular‐weight fragments (<15 kDa), suggesting potential degradation of the cell wall. The addition of NaN_3_ to the BL‐Fe(III) treatment could partially reverse this degradation, as indicated by the less‐intense fragments in the <15 kDa region. LPS is composed of lipid A and sugar subunits. Connecting the lipid A and oligosaccharide chain is the ketodeoxyoctonic acid (Kdo) disaccharide. Kdo, a conserved component across all bacterial LPS, also contains a carboxyl group (Figure 6B, red dashed box). Silver staining revealed that both LPS aggregates (50‐100 kDa) and monomers (10‐20 kDa) substantially diminished after BL‐Fe(III) treatment. This degradation was again reversed by adding NaN_3_ to the reaction. Indeed, fragments smaller than the LPS monomer (<10 kDa) could be detected in the treatment group with extended gel development (Figure 6B, blue dashed box). The degradation of PG by BL‐Fe(III) can lead to cell lysis, akin to the action of penicillin antibiotics, and the damage to LPS could affect membrane integrity, similar to the effect of colistin antibiotics. The result argues strongly for the potential of BL‐Fe(III) to inactivate most of bacterial pathogens by attacking both bacteria itself and the extracellular matrix, regardless of the metabolic state of the bacteria.

### 2.6 BL‐Fe(III) is Effective in Managing Polymicrobial Biofilms in Diabetic Wounds

**Figure 7:**
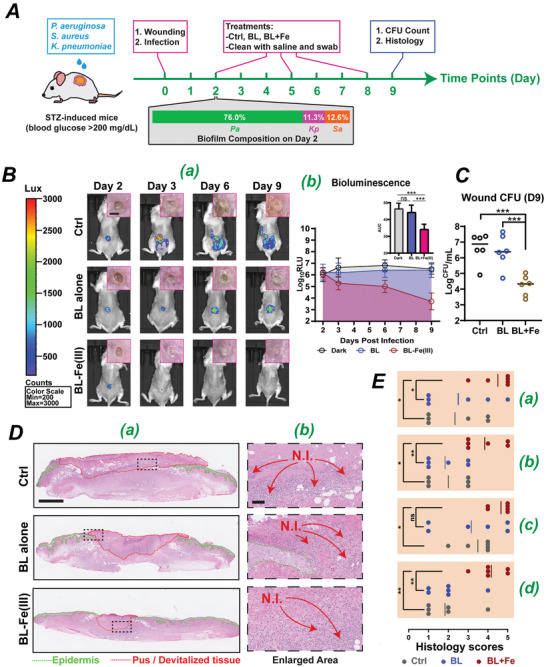
The efficacy of BL‐Fe(III) in treating polymicrobial wound infections in diabetic mice. A) Diagram illustrating the experimental setup for the diabetic mouse study. STZ, Streptozotocin. The wounds were infected by a mixture of *P. aeruginosa* (*Pa*), *S. aureus* (*Sa*), and *K. pneumoniae* (*Kp*) at a ratio of 1:1:1 on day 0, with a total of 1‐5 × 10^5^ CFU per wound. The wounds were subject to three treatments on days 2, 5, and 8. Gray box underneath the timeline indicated the bacterial compositions on day 2 post infection (n = 8). BL, 40 J cm^−2^. Fe(III), 300 µM. B) Bioluminescence images of wound infections over time (a). Insets, photographs of the wounds. Wound bioluminescence plotted over time (b). Inset, area under curve (AUC). n = 6. C) Bacterial loads on day 9 post infection (n = 6). D) H&E staining of wound specimens collected on Day 9. Green dashed lines indicated the areas of epidermis. Red dashed lines highlighted the pus/devitalized tissues. The areas in black dashed boxes in (a) were enlarged in the corresponding panel in (b). N.I., neutrophile infiltration. E) Histology evaluation scores for wound healing. a) Re‐epithelialization; b) Dermis closure; c) Inflammation control and d) Infection control. n = 6. *p ≤ 0.05, **p ≤ 0.01, ***p ≤ 0.001, and ns, no significant difference between indicated groups. Scale bar, 4 mm in (B) inset, 1000 µm in (Da), and 100 µm in (Db).

Chronic wounds, such as diabetic foot ulcers or bedsores, are susceptible to infections caused by multispecies biofilms. These wounds are often infected with multi‐drug resistant (MDR) bacteria and difficult to treat with antibiotics. Repeated cleansing of the wounds is required to reduce the bacterial burden and mitigate the risk of sepsis. However, residual biofilms after cleansing are often still too large for phagocytes to eliminate, leading to persistent neutrophil infiltration, which keeps the wound in the inflammatory phase and delays healing. To assess the effectiveness of BL‐Fe(III) in such cases, a combination of BL at 40 J cm^−^^2^ and Fe(III) at 300 µM that simultaneously killed a significant portion of bacteria and dispersed the biofilm matrix was selected for the animal experiments. We first infected full‐thickness wounds in streptozotocin (STZ)‐induced diabetic mice with three common pathogens: two bioluminescent (Lux) strains, *P. aeruginosa* (*Pa*) 19 660 and *S. aureus* (*Sa*) USA300, along with a wild‐type *K. pneumoniae* (*Kp*) IQ0035 (**Figure** **7**A). By day 2, the multispecies biofilms had been established, with *Pa* dominating (76.0%), followed by *Kp* (11.3%), and *Sa* (12.6%) (Figure 7A, box underneath timeline). The biofilm composition was determined by both colony morphology and Lux signals. Specifically, *Pa* was greenish and Lux positive, *Sa* was yellowish and Lux positive, while *Kp* was white and Lux negative as shown in Figure S9 (Supporting Information). Both *Pa* and *Sa* were engineered strains capable of producing bioluminescence signals. Additionally, Kp colonies were characteristically white and opaque. On days 2, 5, and 8, we administered the designated treatments, gently rinsed the wounds with saline, and used a cotton swab to remove any loosely attached biofilms and superficial contaminants (Figure 7A). Saline was chosen for several reasons: i) it is a common cleansing solution, ii) the monovalent sodium ion in saline can replace divalent ions that cross‐link certain EPS materials, such as alginate,^[^ ^28^ ^]^ making the polymer more soluble and accessible to Fe‐BL treatment, and iii) we demonstrate that polymers like alginate can be easily degraded in the presence of saline (Figure 3A). Due to the bioluminescence of *Pa* and *Sa*, we were able to monitor treatment efficiency in real‐time through Lux signals (Figure 7B,a,b). In the control group, the spread of the wound infection/Lux signal beyond the peri‐wound area over time indicated severe cellulitis (Figure 7Ba, top row). BL alone treatment seemed to effectively confine the infection to the original wound bed, but failed to eliminate the bacteria, as indicated by the persistent Lux signals (Figure 7Ba, middle). In marked contrast, the first BL‐Fe(III) treatment controlled more than 90% bacterial growth, evidenced by a 1‐Log10RLU reduction in Lux signal (Figure 7Bb, day 3 compared to day 2). And repeated BL‐Fe(III) treatments (in total of 3) greatly reduced the Lux signals by 2 Logs on day 9, with some mice showing undetectable levels (Figure 7Ba, bottom row). The total Lux signals, represented by the area under the curve (AUC), in the BL‐Fe(III) treatment group were significantly lower than those in the BL‐alone and control groups (Figure 7Bb, inset). Consistent with the Lux signals, wound images from the control and BL treatment groups displayed severe pus formation. This pus, accompanied by heavy yellow discharges under the wound dressing before cleansing, suggested chronic and non‐healing wound infections. Conversely, wounds that received repeated BL‐Fe(III) treatments showed fewer discharges; the full‐thickness wound beds became increasingly shallow (Figure 7Ba, insets). On day 9, the wounds treated with BL‐Fe(III) had significantly fewer bacteria (4.2 Log^CFU^/wound) than those untreated (6.6 Log) or treated with BL alone (6.4 Log) (Figure 7C, right). It is worth mentioning that bacterial loads maintained at a level of <5 Log^CFU^ per gram are generally considered acceptable and would not delay wound healing.^[^ ^29^ ^]^ Further analysis of the bacterial composition in the wounds revealed that the proportion of skin commensals (i.e., indigenous bacteria other than the inoculated pathogens) in the control, BL alone, and BL‐Fe(III) treatment groups were 4%, 16.5%, and 33%, respectively, suggesting a populational shift toward commensal bacteria after BL and particularly BL‐Fe(III) treatment (Figure S7, Supporting Information). H&E staining of wound tissues on day 9 revealed distinct differences among treatment groups (Figure 7D,E). First, approximately 66% of the wounds treated with BL‐Fe(III) exhibited excellent re‐epithelialization (<1 mm gap, score 5), as indicated by the green dashed lines (Figure 7Da,Ea). The remaining 33% of wounds in the BL‐Fe(III) group exhibited intermediate re‐epithelialization (with a 1–3 mm gap, score 3 or 4). In marked contrast, the control and BL‐alone groups exhibited poor re‐epithelialization, with wide and non‐cover area. Notably, half of the wounds in both groups had non‐epithelialized areas at a diameter larger than 4 mm (score 1), roughly the same size as the original trauma, suggesting that the healing process in these wounds was either halted or deteriorated, likely due to severe biofilm infection (Figure 7Ea). Second, some wounds in the control and BL‐alone groups displayed large areas of pus occupying the wound bed (red dashed lines), accompanied by massive neutrophil infiltration (N.I., marked by red arrows) near the pus, which irritated the surrounding peri‐wound tissues (Figure 7Dab). We assign histology scores to evaluate wound healing in all groups, based on criteria such as the degree of re‐epithelialization (a), dermis closure (b), control of inflammation (c), and infection management (d) (Figure 7E, scoring criteria available in Table S2, Supporting Information). Wounds in the BL‐Fe(III) treatment group showed significantly better healing scores across each criterion with an exception for the inflammation levels, which did not show a significant difference between the BL‐Fe(III) and BL‐alone groups, likely due to wide variation within the BL‐alone group. In marked contrast, the inflammatory area was significantly smaller in BL‐Fe(III)‐treated wounds compared to the untreated control group, suggesting that the dispersed biofilms were less likely to provoke prolonged inflammation (Figure 7Ec) This indicates that the host immune system can effectively control the surviving bacteria within the dispersed biofilm. It is also worth noting that re‐epithelialization consistently precedes dermis closure in healing wounds. For instance, in the BL‐Fe(III) group, three wounds (50%) showed complete re‐epithelialization, yet gaps remained in the dermis (Figure S8, Supporting Information). Rapid re‐epithelialization covers the open wound and protects it from bacterial re‐contamination, thereby allowing the wound healing process to proceed without delay. The bacterial burden observed on day 9 (Figure 7C) at wounds treated with BL‐Fe(III) might represent superficial contamination, which no longer impeded the wound healing trajectory.

## 3 Discussion

Biofilms, covered by exopolymer matrix, are the predominant lifestyle of pathogens, accounting for approximately 65–80% of all microbial infections.^[^ ^30^ ^]^ Tenacious biofilms are found at various infection sites and on medical devices, including wounds, the middle ear, oral cavity, airway, lungs, bladder, heart valves, orthopedic prostheses, and catheters. Notably, wounds are highly susceptible to polymicrobial biofilm infections because they are directly exposed to external contaminants, especially in patients with bedsores or underlying conditions such as diabetes. Polymicrobial biofilms trigger prolonged inflammation and delay or stall wound healing. Chronic wounds require frequent cleansing and periodic debridement to physically remove biofilms, a strategy much like dental hygiene. In dental care, oral biofilms are suppressed through daily brushing and flossing, coupled with periodic dental visits for plaque removal.^[^ ^31^ ^]^ However, current wound hygiene or cleansing solutions such as saline, surfactant, or antiseptic irrigation, do not effectively disrupt biofilms.^[^ ^32^ ^]^ Therefore, standard cleansing is done with as much physical force (e.g., wiping with gauze) as long as the patient can tolerate.^[^ ^33^ ^]^ Even with such painful efforts, biofilms still quickly re‐form and result in wound deterioration between debridement appointments, eventually leading to sepsis or amputations.

In this study, we investigate the potential of BL‐based modality as a convenient and effective option for daily wound cleansing, particularly focusing on the disassembly of biofilm matrix. Disrupting biofilm matrix not only directly reduces bacterial burdens, but also enhances host immune clearance of the microbes and helps reverse antibiotic tolerance associated with biofilms.^[^ ^7^, ^8^ ^]^ However, conventional blue light approaches, which depend on porphyrin to generate ROS, appear ineffective in disrupting the biofilm matrix, as evidenced by the resistance of *K. pneumoniae* capsule polymer to BL alone or BL plus porphyrin treatment (Figure 2). Ferric ions alone have been shown contradictory effects on biofilm formation, but it is clear that ferric ions can bind to biofilms’ EPS, particularly to carboxyl groups.^[^ ^19^, ^34^ ^]^ We combined BL with Fe(III) ion to form Fe(III)‐carboxylate complex, a substrate for blue light‐mediated photolysis.^[^ ^19^ ^]^ The carboxyl groups are ubiquitously presented in a range of matrix biopolymers. Our data revealed that BL‐Fe(III) effectively degraded various EPS of mono‐species or mixed‐species biofilms: it degraded capsule polymer purified from the hypermucoid *K. pneumoniae*, increasing the bacterial susceptibility to macrophage phagocytosis (Figure 2); it broke down alginic acid found in *P. aeruginosa* biofilms, compromising their matrix integrity (Figure 3); BL‐Fe(III) also targeted the complex EPS derived from four wound pathogens and host polymeric materials, indicating broad‐spectral disassembling efficacy (Figure 4).

Current agents for disassembling biofilms include signaling molecules such as D‐amino acids,^[^ ^35^ ^]^ bacterial enzymes like deoxyribonuclease,^[^ ^36^ ^]^ dispersin B,^[^ ^37^ ^]^ and biosurfactants such as rhamnolipids.^[^ ^38^ ^]^ The dispersal mechanism of D‐amino acids remains unclear and controversial.^[^ ^39^ ^]^ On the other hand, enzymes and biosurfactants usually exhibit narrow‐spectrum activity due to their substrate specificity:, i.e., they only disassemble biofilms formed by certain species and under specific conditions. However, biofilms often consist of mixed species. Members of the microbial community and the host each contribute their own EPS, which merge into a complex matrix, similar to the in vitro wound biofilm we have created. Notably, our BL‐Fe(III) treatment also degrades convoluted EPS of polymicrobial biofilms, capable of lysing biopolymers that possess repeated carboxyl groups. Bacterial capsule polysaccharides are an important source of biofilm EPS; in most cases, these capsule polymers contain repeated carboxyl groups, contributing to their negative net charge.^[^ ^18^ ^]^ Besides capsular polysaccharides, many bacterial exopolysaccharides—such as alginate, xanthan, and cepacian—that are released into the surrounding environment, also contain carboxylic moieties owing to the presence of uronic acids and ketal‐linked pyruvate.^[^ ^40^ ^]^ The repeated carboxylic moieties in these polysaccharides make them ideal targets for the BL‐Fe(III) modality. In other words, BL‐Fe(III) can be used to degrade a wide range of biofilm matrices produced by various bacteria.

Apart from the degradation of the extracellular matrix, BL‐Fe(III) can also directly kill a panel of pathogens, irrespective of their tolerance or susceptibility to BL, which is unexpected. We hypothesized and subsequently confirmed that BL‐Fe(III) targeted essential subunits in bacterial surface structures, such as N‐acetylmuramic acid (NAM) and 3‐deoxy‐D‐manno‐octulosonic acid (Kdo). These subunits are crucial for the cell walls and outer membranes of bacteria, respectively, and are rich in carboxylic groups. By targeting these specific subunits, BL‐Fe(III) demonstrates a mechanism of action akin to penicillin‐mediated disruption of cell wall synthesis, and colistin‐like antibiotics that impact the outer membrane, ultimately leading to bacterial death. Therefore, the BL‐Fe(III) modality is superior to regular biofilm dispersal agents because it not only disperses a broad range of matrices but also directly inactivates the embedded bacteria. In contrast, dispersal agents merely degrade the matrix, leaving the bacteria alive. We demonstrated that BL‐Fe(III) treatment, combined with repeated wound cleansing, resulted in significantly better healing outcomes in diabetic wounds co‐infected with *P. aeruginosa*, *S. aureus*, and *K. pneumoniae* (Figure 7).

A notable limitation of the BL‐Fe(III) modality is the poor skin penetration of blue light.^[^ ^41^ ^]^ Therefore, it is not our intention to replace the conventional sharp debridement with the BL‐Fe(III) treatment, especially for severe and deeply infected wounds, because debridement can remove devitalized tissues and encourage wound healing. This approach is advantageous because the disassembly of the biofilm by BL‐Fe(III) may increase the surface‐to‐volume ratio of the bacterial population and facilitate the regrowth of surviving bacteria, rendering them more susceptible to antibiotics, as most antibiotics target actively growing cells.

The BL‐Fe(III) regimen is an innovative, cost‐effective alternative that complements current strategies for cleansing biofilm‐infected wounds. It can be performed daily, much like brushing and flossing in dental care. Wearable and stretchable BL LED patches are affordable and ferric ions are also cost‐effective. Together, they offer significant benefits for patients managing chronic wounds at home, particularly in areas with limited healthcare resources. Moreover, while conventional debridement is effective in reducing bacterial load and breaking biofilms, it is often painful and impractical for home use. In contrast, the BL‐Fe(III) modality has the potential to serve as a safe and simple photo‐debridement tool for wound cleansing. It offers a non‐painful alternative for managing both acute and chronic wound infections, including diabetic ulcers, pressure sores, venous ulcers, post‐surgery infections, and necrotizing fasciitis.

## 4 Experimental Section

### Light Source, Fe(III), and Microorganisms

A light‐emitting diode (Thorlabs) with peak emission at 405 nm and a full width at half maximum of 12.5 nm was used. The irradiation was adjusted to 55 mW cm^−2^. Fe(III) chloride hexahydrate, FeCl₃·6H₂O, was purchased from Thermo Fisher Scientific. A 100 mM Fe(III) stock solution was prepared in deionized water and subsequently diluted with saline. The strains and culture conditions used in this study are listed in Table S1 (Supporting Information).

### BL‐Fe(III) Treatment on Hypermucoid K. pneumoniae Culture

A hypermucoid colony was cultured overnight in BHI broth. A 1 mL aliquot of the viscous culture was transferred to a 48‐well plate and treated with blue light at 80 J cm^−^^2^, or left untreated. The bacterial culture was transferred to a 1.5 mL Eppendorf tube and centrifuged at 10 000 rpm for 2 min. After centrifugation, the supernatant (upper layer) was transferred to a 96‐well plate to measure the optical density at 595 nm.

### Extraction of Capsule Material from Hypermucoid K. pneumoniae

The capsule material was extracted using the hot phenol method, as previously described, with some modifications.^[^ ^42^ ^]^ Briefly, the hypermucoid *K. pneumoniae* was spread on BHI agar (9‐mm dish) and allowed to grow overnight. The sticky bacterial lawn was carefully scraped into a Falcon tube, washed once with 5 mL of distilled water, and then resuspended in 500 µL of distilled water. The suspensions were incubated for 2 min at 68 °C, 500 µL of phenol was added, and the samples were incubated for an additional 30 min at 68 °C. After incubation, 500 µL of chloroform was added to the suspension, which was then centrifuged at 12 000 rpm for 5 min to separate and extract the aqueous layer. Three volumes of ethanol were added to the suspension to precipitate the polysaccharides, and the sample was stored at ‐20 °C overnight. The polysaccharides that had precipitated were collected by centrifugation at 12 000 rpm for 30 min, after which the ethanol was removed. After extraction, the polysaccharides were dissolved again in 0.8% NaCl, 0.05% NaN3, and 0.1 M Tris‐HCl (pH 7). The solution was subsequently incubated with 50 µg mL^−1^ of DNase II type V and RNase A for 18 h at 37 °C. This was followed by protein digestion using 50 µg mL^−1^ of Proteinase K and incubating for 1 h at 55 °C followed by 24‐h incubation at room temperature. The polysaccharides were precipitated by adding five volumes of methanol containing 1% (vol/vol) saturated sodium acetate, followed by overnight incubation at ‐20 °C. After drying, the materials were weighed. The sample was then dissolved again in water.

### Staining of Biopolymers

The integrity of the biopolymers following BL‐Fe(III) treatments was analyzed using 4–15% Mini‐gel electrophoresis (Bio‐Rad). Carboxyl‐containing polysaccharides, such as the *K. pneumoniae* capsule and alginic acid, were stained by the positively charged Alcian Blue dye.^[^ ^42^ ^]^ In brief, the gel was rinsed six times with ultrapurified water, stained with 0.1% alcian blue for 60 min, and then destained overnight using a solution of 60% 20 mM sodium acetate (pH 4.75) and 40% ethanol. The container was covered with plastic wrap to prevent ethanol evaporation and ensure effective destaining. Other biopolymers, such as starch (Sigma), peptidoglycan (PGN from *S. aureus*, Invivogen), and lipopolysaccharides (LPS from *E. coli*, Sigma), were stained using the silver staining method, with modifications.^[^ ^43^ ^]^ Briefly, the gel was first oxidized with 0.7% periodic acid in a 40% ethanol‐5% acetic acid solution at 22 °C for 20 min, without prior fixation. The treated gel was then sensitized and stained according to the standardized protocol (Thermo Scientific).

### Transmission Electron Microscopy (TEM)

To preserve the structures of capsular polysaccharides, colonies of *K. pneumoniae* were scraped from BHI agar in a 9‐mm dish, then soaked in either saline or a 300‐µM Fe(III) solution before being irradiated with blue light at 80 J cm^−^^2^. The bacterial aggregates, whether treated or untreated, were immediately fixed in a solution of 2.5% glutaraldehyde and 2% paraformaldehyde, then prepared for TEM as previously described.^[^ ^44^ ^]^ The sections were examined on a Philips CM‐10 TEM (Eindhoven, The Netherlands).

### Macrophage‐Mediated Phagocytosis

*K. pneumoniae* strains in the log phase were diluted in either saline or a 300‐µM Fe(III) solution. The saline‐diluted samples were kept in dark, while the Fe(III)‐diluted samples were irradiated with blue light at 80 J cm^−^^2^. Macrophage RAW 264.7 cells were infected with the untreated or treated *K. pneumoniae* at a multiplicity of infection (MOI) of 100 for 2 h. Extracellular bacteria were eliminated by washing three times, and the infected macrophages were then cultured with gentamicin (200 µg mL^−1^) for another hour. Internalized bacteria were visualized using a Live/Dead staining kit (Thermo Fisher Scientific) and a confocal laser scanning microscope (FV1000, Olympus), as previously described.^[^ ^12c^ ^]^ Macrophage‐mediated phagocytosis was quantified by the percentage of activated macrophages (i.e., Mφ^+^/Mφ ratio) in each group. An activated macrophage (Mφ^+^) was identified as having engulfed at least one bacterium. The Mφ^+^/Mφ ratio was determined by analyzing 15 random fields of view (FOVs) across three independent experiments.

### BL‐Fe(III) Treatment on P. aeruginosa Aggregates and Biofilms

The slimy *P. aeruginosa* ATCC 19 660 was cultured in a 29‐mm dish with a glass bottom (Cellvis) for two days. The bacterial aggregates were transferred to new 3‐mm dishes and subjected to different treatments (i.e., dark control, 40 J cm^−^^2^ blue light (BL), BL‐Fe, and BL‐Fe‐NaN₃). The treated bacterial aggregates were pipetted up and down 20 times to disperse the entangled cells. Biofilms attached to the original dishes were subjected to the same four treatments. The treated biofilm samples were gently washed three times and then visualized using either 2% crystal violet staining or a Live/Dead staining kit (Thermo Fisher Scientific) with a confocal laser scanning microscope (FV1000, Olympus).

### BL‐Fe(III) Treatment on Polymicrobial Biofilms

As previously described, in vitro polymicrobial biofilms were grown in Lubbock wound‐like medium containing 48% Bolton Broth (NEOGEN), 50% Bovine Plasma (HemoStat), and 2% Laked Horse Blood (HemoStat).^[^ ^24^ ^]^ Briefly, a bacterial mixture containing *P. aeruginosa*, *S. aureus*, *E. coli*, *K. pneumoniae*, and *A. baumannii*, each at 10⁵ CFU, was added to 6 mL of wound‐like medium; a 1 mL pipette tip (Fisher Scientific) was provided as a surface for biofilm attachment. The cultures were incubated at 37 °C with slow shaking at 100 rpm for 48 h. The formed biofilm was peeled off the pipette tip and cut into small pieces, each measuring 3 × 3 mm. The biofilm samples were soaked in various Fe(III) solutions (0, 100, and 1000 µM in saline) and subsequently treated with increasing dosages of blue light. After irradiation, the samples were pipetted up and down 20 times to disperse any compromised biofilm material.

### Cytotoxicity

Human skin fibroblasts were submerged in saline supplemented with 10% FBS and a range of Fe(III). The cells were kept in the dark or exposed to 40 J cm^−^^2^, which is equivalent to 12 min of irradiation. The treated cells were incubated in DMEM overnight to allow for the completion of the apoptotic process, if any, before cell viability was assessed by the CCK‐8 assay according to the manufacturer's protocol (Apexbio Technology).

### Antimicrobial Assay of BL‐Fe(III)

Overnight cultures of *S. aureus*, *E. faecalis*, *K. pneumoniae*, *E. coli*, and *P. aeruginosa* were diluted with saline to obtain a concentration of ≈10^7^ CFU mL^−1^. The bacterial suspensions were kept in the dark, treated with 300 µM Fe(III), exposed to 40 J cm^−^^2^ blue light, or subjected to a combination of blue light and Fe(III). Blue light irradiations were performed in a transparent 48‐well plate (Corning). Surviving bacteria were determined using a microdilution plating method, with CFU counts having a detection limit of 100 CFU mL^−1^.

### Diabetic Animals, Polymicrobial Wound Infection, and Treatment of the Wound Infection

BALB/c mice, aged 8 weeks, were purchased from Charles River Laboratories. All animal procedures, conducted under protocol number 2020N000077, were approved by the Institutional Animal Care and Use Committee of Massachusetts General Hospital in accordance with National Institutes of Health guidelines. Induction of type I diabetes in female BALB/c mice was achieved as previously described.^[^ ^45^ ^]^ Briefly, streptozotocin (STZ, 200 mg kg^−1^, Sigma) dissolved in sodium citrate buffer (pH 4.0) was administered via intraperitoneal injection. To mitigate potential hypoglycemia, 10% sucrose was provided in the drinking water for the first 72 h following the STZ injection. Glucose levels of the mice were monitored by a Blood Glucose Meter (Germaine Laboratories) for three consecutive days after one week of STZ injection and one day before wounds were created. Mice exhibiting glucose levels over 200 mg dL^−1^ for all four tests were confirmed diabetic and were enrolled in the study. A full‐thickness wound was created on each diabetic mouse by a 4‐mm biopsy punch (Precision Medical Devices). The wounds were co‐infected with *S. aureus* USA300 LAC, *K. pneumoniae* strain IQ0035, and *P. aeruginosa* ATCC 19 660 Lux (1‐5 × 10^5^ CFU per wound) for two days to establish a polymicrobial infection. The infected diabetic mice were separated into three groups: (i) no treatment (control), (ii) treatment with blue light alone, and (iii) BL‐Fe(III) combination treatment. Repeated treatments were administered on Days 2, 5, and 8, respectively. All mice received basic wound cleansing after treatments. Bioluminescence monitoring of the wound infections was done one day after each treatment, i.e., on Days 3, 6, and 9. Mice were euthanized on Day 9 for tissues collection and wound bacterial enumeration.

### Histological Analyses

The diabetic wound tissues collected on Day 9 post‐infection were subjected to histological examination and scoring. The sections were visualized by NanoZoomer 2.0 HT (Hamamatsu) and the images were analyzed by NDP viewer software (Hamamatsu). Scoring criteria are listed Table S2 (Supporting Information). Each group had six biological replicates.

### Statistical Analyses

All statistical analyses were performed using GraphPad Prism 10. Statistics significance was analyzed with two‐tailed Student's t‐test between two groups. P values of <0.05 were considered significant. Data were presented as means ± SDs.

## Conflict of Interest

The authors declare no conflict of interest.

## Author Contributions

M.X.W. and Y.L. performed conceptualization. M.X.W. and Y.L. performed methodology. Y.L., Y.D., Z.Z., and C.L. performed investigation. Y.L. and Z.T.L. performed visualization. M.X.W. and Y.L. performed funding acquisition. M.X.W. performed project administration. M.X.W. performed supervision. M.X.W. and Y.L. performed writing – original draft. M.X.W. and Y.L. writing – review and editing.

## Supporting information



Supporting Information

Supplemental Video 1

Supplemental Video 2

Supporting Information

## Data Availability

The data that support the findings of this study are available from the corresponding author upon reasonable request.
