# Supplementary material for: Efficient Photolysis of Multidrug‐Resistant Polymicrobial Biofilms
Source: Adv Sci (Weinh). 2024 Dec 21;12(6):2407898. doi: 10.1002/advs.202407898 (PMC11809414; doi:10.1002/advs.202407898)
Supplement: Supplementary file 1 — Supporting Information [file ADVS-12-2407898-s001.docx]

**Efficient Photolysis of Multidrug-Resistant Polymicrobial Biofilms**

**Supplementary Figures and Tables**

**
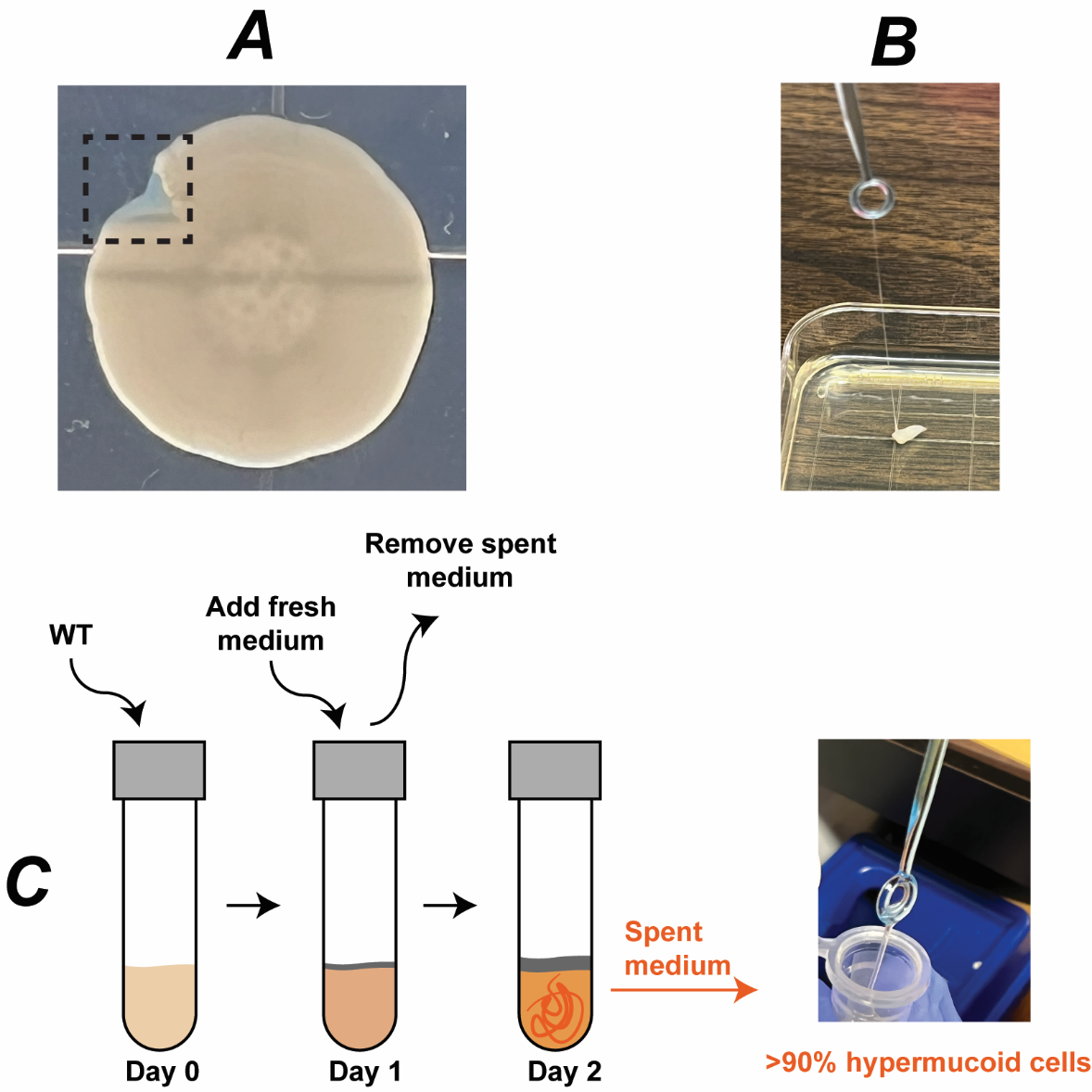
**

**Fig. S1. Spontaneous mutation of *Klebsiella pneumoniae*.** (**A**) The non-capsulated mutant spontaneously rose when the wild-type(WT) was cultured on agar for three days. The black dashed box highlighted the translucent non-capsulated mutant emerged from a WT colony. (**B**) The hypermucoid mutant was positive for string test. A long string (> 5 mm) could be drawn from the slimy colony. (**C**) Illustration of the culturing method that encouraged biofilm growth and the rise of hypermucoid mutant. The resulting liquid culture on Day 2 was very viscous and contained more than 90% of hypermucoid cells, determined by CFU count.

**Fig. S2. *Klebsiella pneumoniae* strains are resistant to blue light inactivation.** The non-capsulated and hypermucoid mutants remain resistant to blue light inactivation, similarly to the wild type K. pneumoniae. n=4. BL, 80 J/cm^2^.

**
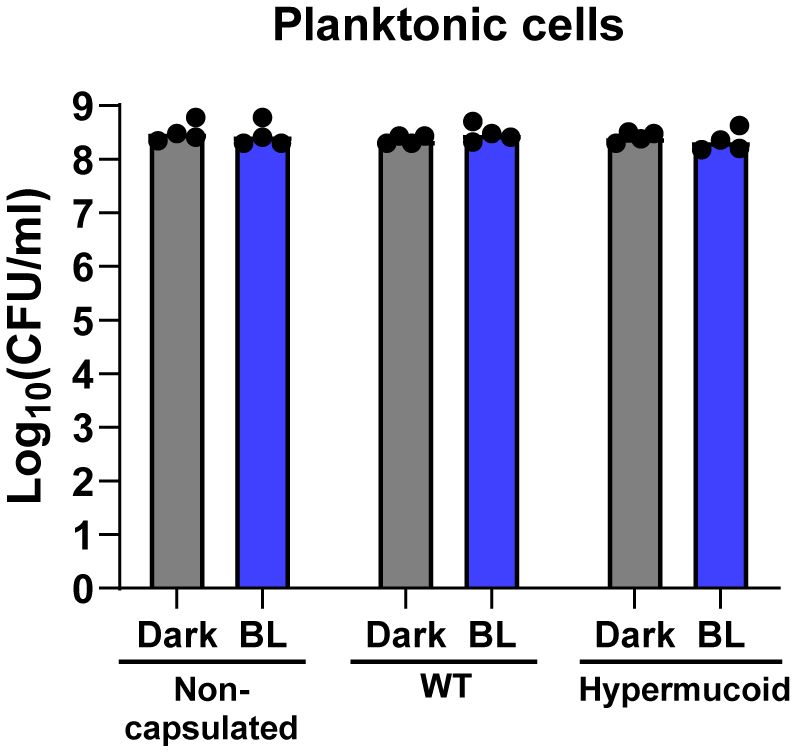
**

**
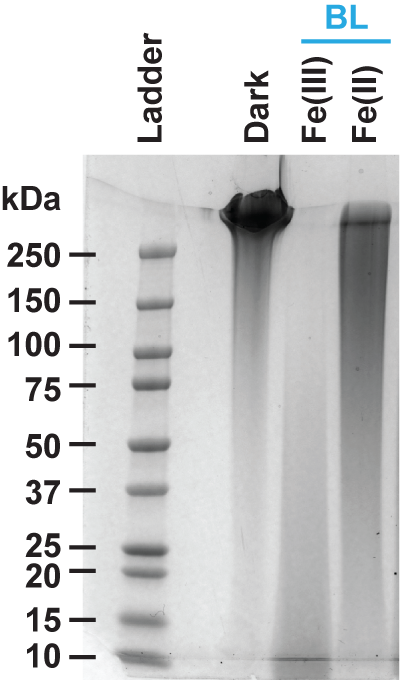
**

**Fig. S3. BL-Fe(III) is more potent than BL-Fe(II) in degrading alginate polymers.** The negatively charged alginic acid was stained by Alcian Blue. Upon blue light (BL) irradiation, Fe(II) catalyzes a limited breakdown of the polymer, whereas Fe(III) facilitates rapid degradation. Fe(III) and Fe(II), 1000 µM. Alginic acid, 10 µg/Lane. BL, 100 J/cm^2^.

**Fig. S4. BL-Fe(III) is unable to degrade non-carboxylate polymer: starch.** Starch is composed of D-glucose subunits, which do not contain carboxylic acid. The starch (10 µg/lane) was visualized using silver staining. No additional smears or bands of smaller sizes were identified in the BL-Fe(III) treatment group compared with the BL alone or the Dark control groups. BL, 100 J/cm^2^.


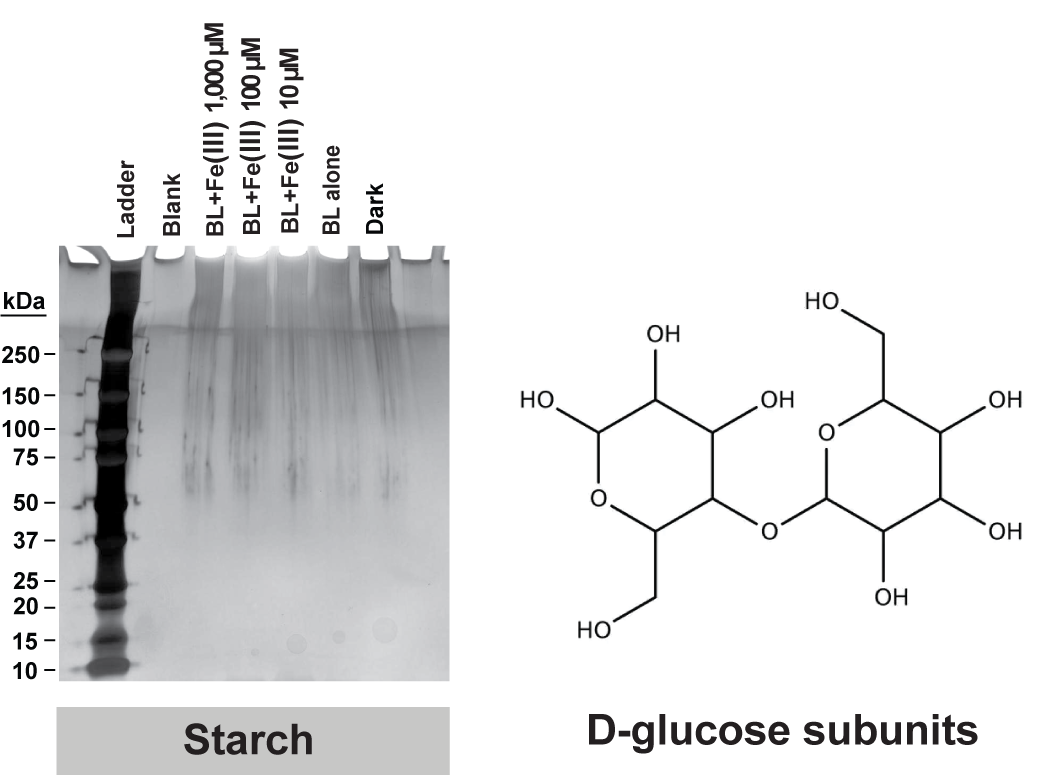


**Fig. S5. BL-Fe(III) compromise the structural integrity of polymicrobial biofilms.** The polymicrobial biofilm consists of five common wound bacteria, including P. aeruginosa, K. pneumoniae, S. aureus, A. baumannii, and E. coli. (**A**) Biofilm treated by blue light alone. The biofilm is resistant to mechanical disruption, like pipetting up and down. (**B**) Biofilm treated by the BL-Fe modality. The biofilm becomes fragile after BL-Fe(III) treatment. BL, 20J/cm^2^. Fe(III), 1000 µM.

**Please find the attached video clips (A and B) in supplementary information.**

**
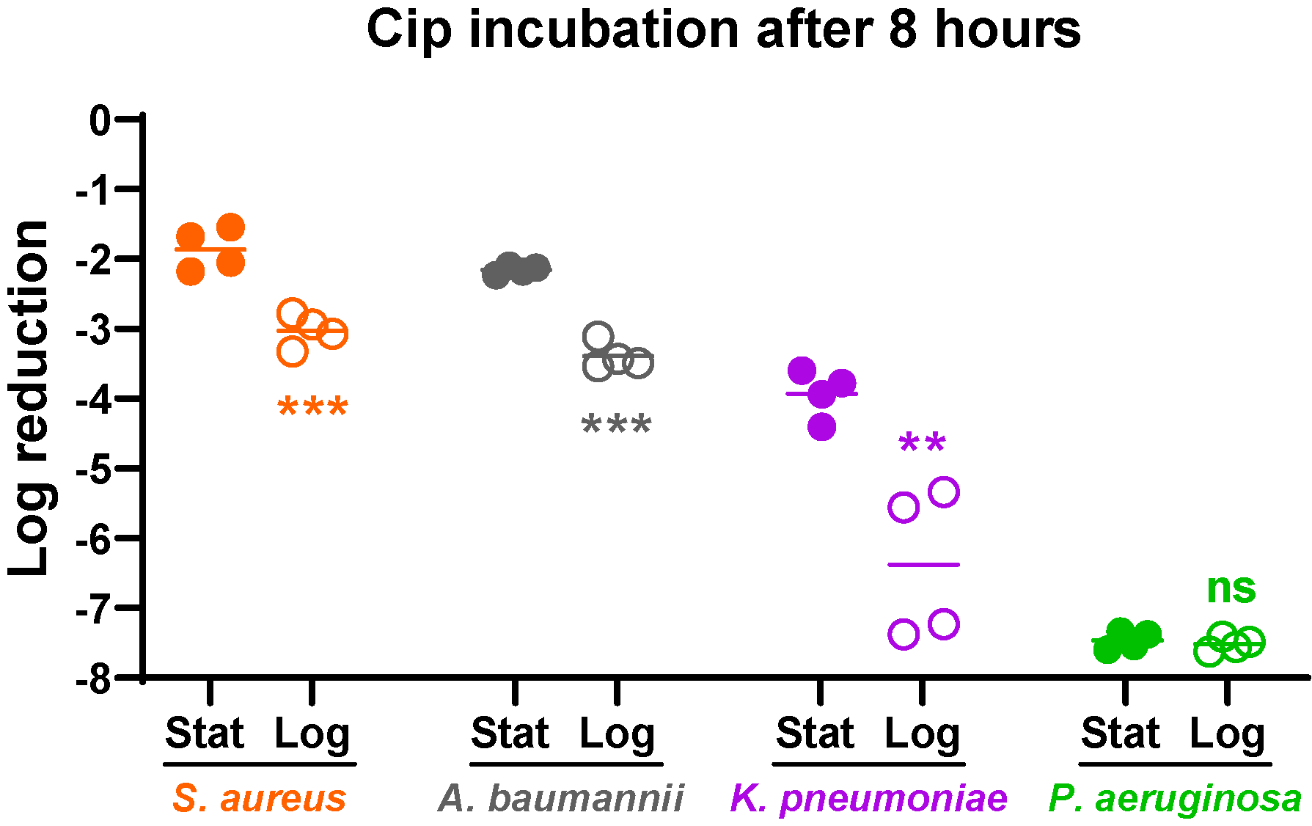
**

**Fig. S6. The tolerance of wound bacteria to ciprofloxacin at different growth phases.** Wound pathogens, except P. aeruginosa, in the stationary (Stat) phase showed higher tolerance to the bactericidal drug ciprofloxacin (Cip) than their counterparts in the log phase. Bacteria were incubated with 10 µg/mL Cip for 8 hours.


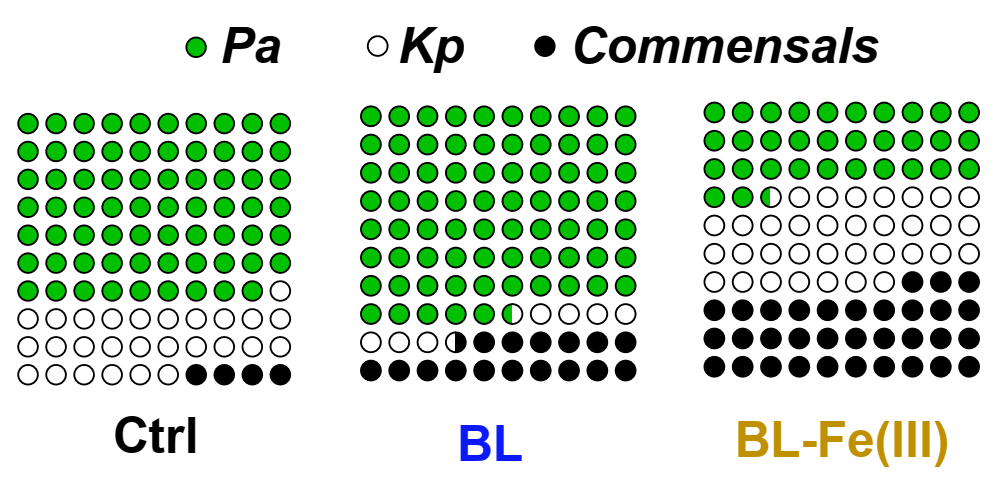


**Fig. S7. Bacterial species composition in diabetic wounds on Day 9 after various treatments.** Each circle represents 1% of the total bacterial population. The diabetic wounds were initially infected by P. aeruginosa (Pa), K. pneumoniae (Kp), and S. aureus (Sa). Both Pa and Sa are engineered strains capable of producing bioluminescence signals. Additionally, Kp colonies are characteristically white and opaque. Other bacterial colonies recovered from the wounds, which were neither bioluminescent nor white, were considered skin commensals of the mice. A clear trend observed was the significant increase in commensal bacteria at the wound site following BL-Fe(III) treatments. It is probable that the BL-Fe(III) treatment eradicated a majority of the Pa, Kp, and Sa pathogens, creating vacant niches around the wound that were quickly occupied by nearby commensals. The increasing population of skin commensals indicates a trajectory towards wound healing. Fe(III), 1000 µM. BL, 100 J/cm^2^.

**
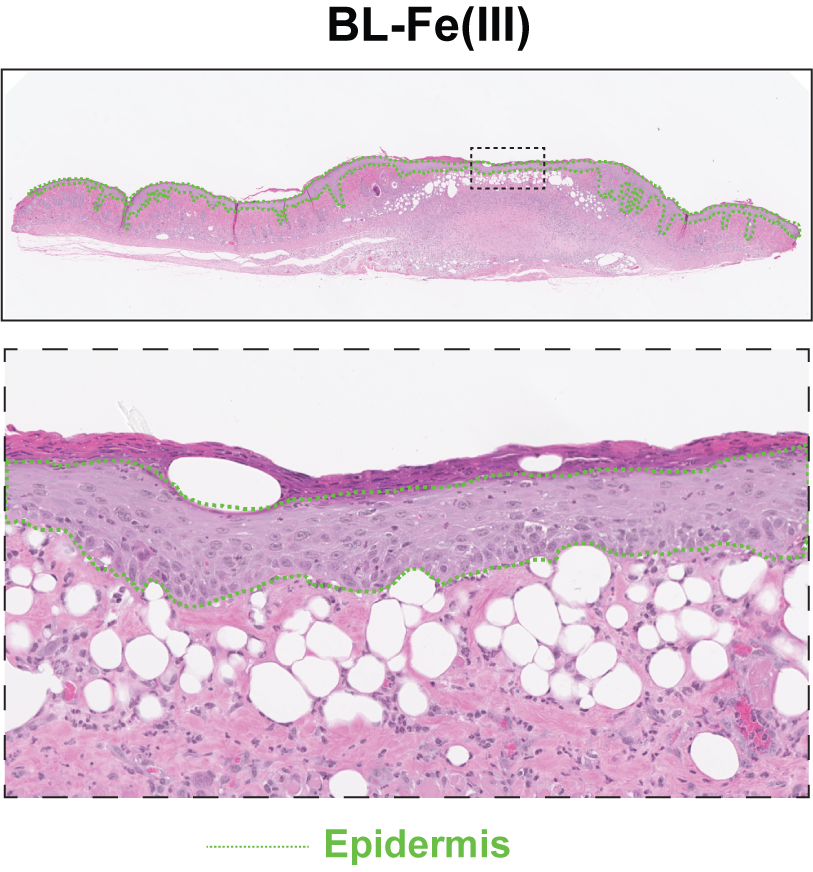
**

**Fig. S8. A representative showing BL-Fe(III) promotes complete re-epithelization in some diabetic wounds.** Green dashed area indicated the epithelia layer of the mouse skin. The enlarged dashed box highlights the complete re-epithelialization at the wound site, where the epithelium directly connects with the underlying fat tissues, and the dermis layer has not yet regenerated. The bump under the dashed box indicates inflammation, which is due to the infiltration of neutrophils and macrophages, suggesting the possibility of a remaining bacterial infection and/or tissue remodeling.

**
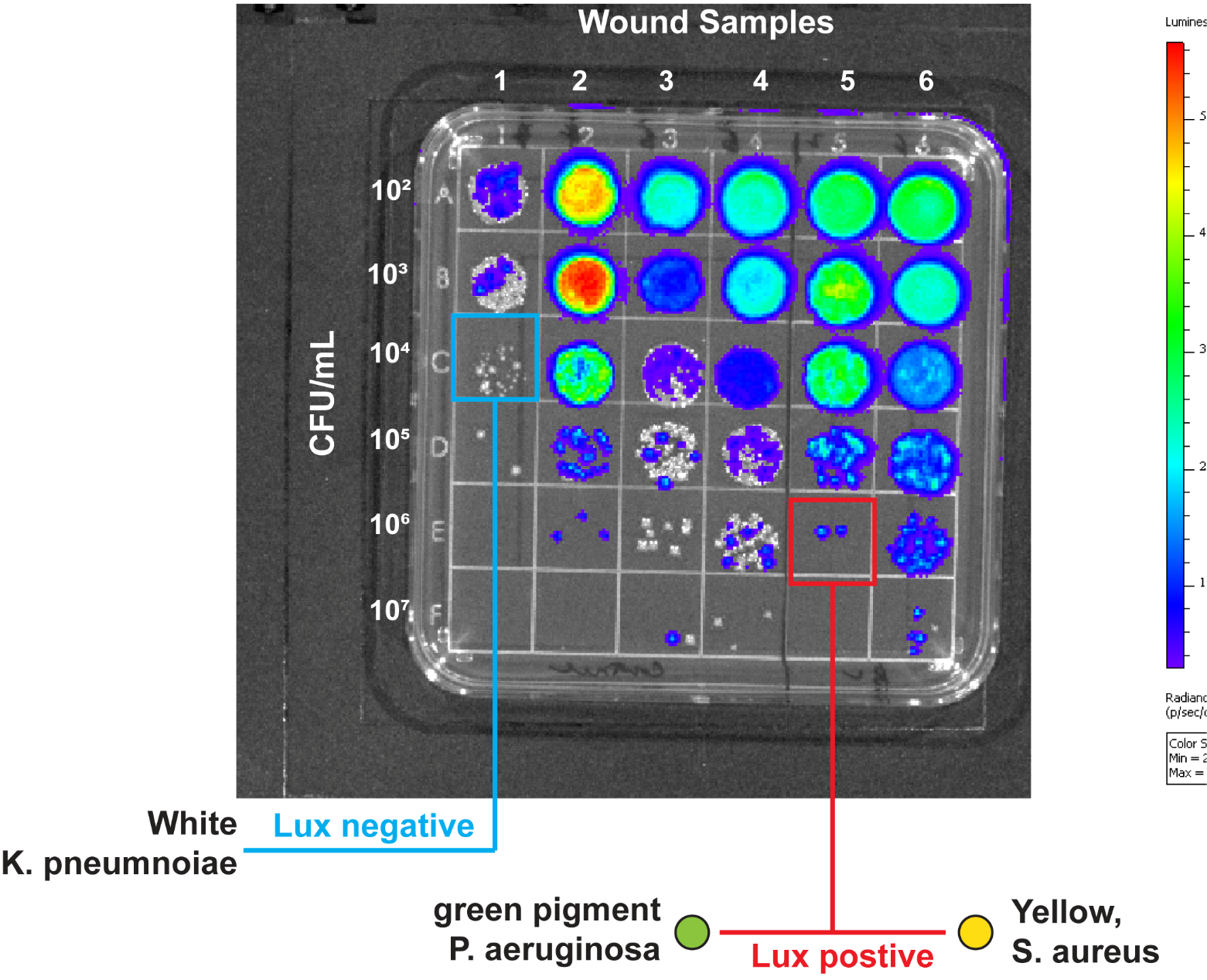
**

**Fig. S9. A representative IVIS image showing how different bacterial species were differentiated on agar plate.** Bioluminescence (Lux) signals of the bacteria on agar plate were first examined by under IVIS (In Vivo Imaging System, PerkinElmer). Lux-positive bacteria were further examined by the colony morphology and color—S. aureus produces yellow pigment. P. aeruginosa produce green, sometimes brown pigment. Lux-negative bacteria were mainly K. pneumoniae.

**Table S1. Bacterial strains and culturing conditions.**

**Table S2. Scoring criteria for histological analysis.**
